# Hepatitis C Virus Seroprevalence and Genotypic Distribution Among Hemodialysis Patients at a Teaching and Training Hospital in Western Rajasthan, India

**DOI:** 10.7759/cureus.67175

**Published:** 2024-08-19

**Authors:** Shivani Khullar, Varun Kothari, Prabhat K Khatri, Tejaswee Lohakare

**Affiliations:** 1 Microbiology, Dr. Sampurnanand Medical College, Jodhpur, IND; 2 Child Health Nursing, Srimati Radhikabai Meghe Memorial College of Nursing, Datta Meghe Institute of Higher Education & Research, Wardha, IND

**Keywords:** hcv genotypes, seroconversion, line probe assay, elisa, hepatitis c virus

## Abstract

Background

Hepatitis C virus (HCV) infection is a chronic hepatotropic blood-borne infection. The transmission of HCV in patients undergoing hemodialysis (HD) is more common in comparison to the general population due to factors such as frequent blood transfusions, prolonged vascular access, and the potential for nosocomial infections. Western Rajasthan in India is home to numerous teaching and training hospitals that cater to a large number of HD patients. Understanding the seroprevalence and genotypic distribution of HCV in this specific patient population is crucial for assessing the extent of infection within this vulnerable group for targeted surveillance and developing effectively tailored treatment protocols in healthcare settings. Hence, this study was conducted with an aim to determine seroprevalence, seroconversion, and genotypes of HCV in HD patients at a tertiary care hospital.

Methods

This was a cross-sectional observational study. The duration of the study was from July 2019 to March 2022. In this study, the patients undergoing maintenance HD due to chronic kidney disease (CKD) were recruited. The data collected include patients’ demographics, etiology of CKD, underlying other co-morbidities, duration of dialysis, and biochemical and blood count parameters. The patients recruited at the start of the study were screened for anti-HCV antibodies by HCV enzyme-linked immune sorbent assay (ELISA). The anti-HCV antibody-negative patients were followed up for the detection of anti-HCV antibodies. At the end of the follow-up period, all anti-HCV antibody negative samples in the pool of five and all anti-HCV antibody positive samples were subjected to a real-time polymerase chain reaction (RT-PCR) of 5′ untranslated region (5’UTR) and core region, followed by line probe assay (LiPA).

Results

In this study, after applying inclusion and exclusion criteria, a total of 109 patients were recruited, out of which 64 (58.7%) were males and 45 (41.3%) were females. The age range of participants was 11-88 years with a mean and standard deviation of 46.75 and 16.35 years, respectively. A total of 39 patients (20 on screening, 19 on follow-up) were detected anti- HCV antibody positive. By RT-PCR, 24 patients tested HCV RNA positive (10 on screening, 14 on follow-up). Among 24 HCV RNA-positive samples, LiPA showed, HCV genotype 1a (n=21), genotype 3b (n=1), and two samples were detected to be inconclusive.

Conclusion

The increasing duration of dialysis is significantly associated with acquiring HCV infection. The majority of the cases of CKD in this geographical region are due to hypertensive nephropathy. There can be discordance between antibody and viral RNA positivity in HCV infection. The predominant HCV genotype identified in the dialysis ward of tertiary care hospital was genotype 1a.

## Introduction

Hepatitis C virus (HCV) infection is a global health problem, with an estimated disease burden of more than 58 million people chronically infected, with about 1.5 million deaths occurring per year [1]. Factors such as blood transfusion, renal transplantation, and treatment with hemodialysis (HD) are the main culprits for HCV infection among HD patients [2]. Use of unsterilized injection needles, total transfused blood volume, repeated blood transfusions, improper enforcement of universal precautions, prolonged vascular access, contact with contaminated patients and equipment, violations of infection control procedures such as proper cleaning and disinfection of environmental surfaces and equipment, hand hygiene, personal protective equipment, high co-morbid illness burden, and more frequent interventions are among the factors that contribute to HCV infection during HD [2]. Patients on maintenance HD are at higher risk of acquiring HCV infection [3]. HCV prevalence in patients on maintenance HD spans from 6% to 60%, while studies from India reported prevalence ranging from 4.3% to 45% [4,5].

Data obtained in France, Germany, Italy, Japan, Spain, the United Kingdom, and the United States between 1998 and 2001 for the Dialysis Outcomes and Practice Patterns Study (DOPPS) revealed a mean HD center prevalence of HCV infection of 13.5% and a 9.5% seroprevalence among HD patients in 12 nations enrolled from 1996 to 2011 [6,7]. HCV is a hepatotropic, enveloped, single-stranded, positive sense RNA virus belonging to the Flaviviridae family. Its genome consists of approximately 9,600 nucleotides in length. The HCV has four structural proteins consisting of the core (C), envelope 1 (E1), envelope 2 (E2), and transmembrane (p7) proteins. The six nonstructural (NS2 to NS5B) proteins of HCV are essential to viral replication and growth and therefore are attractive targets for antiviral therapy. The HCV genome is characterized by seven major genotypes that are further divided into 86 subtypes based on phylogenetic analysis of a large number of nearly full-length sequences. The major genotypes are named HCV genotype 1 to genotype 7 [8]. Despite the economic status, genotypes 1 and 3 predominate in the majority of different countries, whereas genotypes 4 and 5 are commonly found in lower-income nations. With 83.4 million cases (46.2% of all HCV cases), genotype 1 is the most widespread globally; almost one-third of these instances are in East Asia [9]. HCV genotype 1 is also reported exclusively among HD patients from the southern part of India [10]. The second most common genotype in the world (54.3 million, or 30.1%) is genotype 3. Genotypes 2, 4, and 6 account for 22.8% of all cases while other genotypes make up the remaining <1% [9].

Now direct-acting antivirals (DAAs) are advised irrespective of HCV genotype, but before the advent of DAAs, genotypes were required for the treatment of HCV infection. The significance of genotype detection is for epidemiological purposes and in the management of cases where simplified treatment with DAAs cannot be prescribed such as resistance-associated substitutions. HCV genotyping aids in the tailoring of treatment to individual patients, since various regimens may be more effective or have a higher barrier resistance for particular genotypes. It also helps in determining disease severity and directs monitoring and treatment, as HCV genotype 3 is associated with severe liver disease and fibrosis, and genotype 3 requires prolonged or alternative treatment. While DAAs have eased HCV therapy, HCV genotyping is still required for personalized medicine, treatment optimization, and public health initiatives [11]. There are various molecular techniques to detect HCV genotypes, which include gold standard test gene sequencing and DNA hybridization tests (i.e., line probe assay (LiPA), polymerase chain reaction-restricted fragment length positioning (PCR-RFLP), type-specific PCR, and nested PCR. This study aimed at the detection of seroprevalence of HCV and its 76 genotypes in an HD ward at a tertiary care hospital.

## Materials and methods

Study design and setting

It was a cross-sectional observational study. The Strengthening the Reporting of Observational Studies in Epidemiology (STROBE) guidelines were used for reporting and preparing the manuscript. The study was conducted in the Department of Microbiology and the Department of Nephrology of Dr. Sampurnanand Medical College of Western Rajasthan, India. The study duration was from July 2019 to March 2022. This study was approved by the Institutional Ethics Committee of Dr. Sampurnanand Medical College, Jodhpur vide Letter No: SNMC/IEC/2019/09. Date: 21.01.2019. A written informed consent was taken from every patient.

Selection criteria

Patients who were undergoing maintenance hemodialysis due to CKD with a minimum of six months of maintenance HD, those with negative HCV antibody tests at the time of screening (for incidence rate), and those who gave written informed consent were included in the study. The patients who refused to give consent to participate in the study, patients with short-term dialysis due to acute renal failure (ARF), HCV-negative patients who were lost to follow-up, and those with less than six months of maintenance HD were excluded from the study.

Data sources and measurement of variables

Data such as patient’s demography, etiology of CKD, current complications, duration of hemodialysis, and number of blood units transfused were collected from patients’ files. History related to risk factors of HCV infection and family history of HCV infection in immediate family members was also taken. All these data were collected in patient proforma at the site. For the diagnosis of HCV infection, whole blood sample from patients was collected by venipuncture in plain/clot activator and ethylenediaminetetraacetic acid (EDTA) gel separator blood vials separately. Samples were transported to the immunology laboratory without any delay and screened for anti-HCV antibodies. The testing algorithm recommended by the Centre for Disease Control and Prevention (CDC) was adopted [12]. At the start of the study, we screened all the patients for the presence of anti-101 HCV antibodies in the serum via the enzyme-linked immune sorbent assay (ELISA) method using Merilisa HCV (Meril Diagnostics, Vapi, India). The patients who were negative for anti-HCV ELISA were followed up every six months for the presence of anti-HCV antibodies. At the end of the study period, blood sample in EDTA vials of all anti-HCV Ab-negative patients was also collected, and all were subjected to pool testing of the optimal pool size of five samples for the detection of HCV RNA as per the study conducted by Saldanha et al. [13]. The plasma samples were stored at -70°C for further genotyping.

Nucleic Acid Extraction and RT-PCR Test

The plasma samples (39 individual samples and 14 pools) were tested for the RT-PCR of 5’UTR and core regions of HCV RNA. Viral RNA extraction was processed by using a “Viral DNA/RNA Extraction kit” from NLM (Nuclear Laser Medicine, Settala MI, Italy) Diagnostics. The HCV RNA 5’-UTR region was chosen as the target to detect the HCV presence in human plasma samples because it is highly conserved among HCV genotypes. Co-amplification of the core region is included in order to discriminate genotypes 1a, 1b, and 6 in the case of samples that have to be analyzed by genotyping. Synthetic RNA provided with the kit was used as the positive control, while the negative control used was human plasma non-reactive for HIV and HCV antibodies and RNA HBS antibodies and DNA. The internal control (IC) was endogenous (mRNA of the housekeeping gene GAPDH) and co-extracted and co-amplified in order to monitor the complete procedure. Viral RNA retro-transcribed and amplified in one-step RT-PCR real-time instrument CFX96 (Bio-Rad Laboratories, Inc., Hercules, CA) using appropriate enzymes and buffer conditions. NLM HCV genotyping kit GEN-C 2.0 code AC004 from NLM Diagnostics was used for the real-time RT-PCR detection of HCV RNA. The amplification mix used consists of two specific probes (for the IC and for the HCV 5’-UTR region). Each probe was labeled with a fluorescent reporter dye (different for both probes) and a quencher dye. In the presence of a light source, the fluorescence emitted by the donor fluorophore is absorbed by the proximal quencher. During the amplification, each probe matches its specific sequence, and it is the target of the 5 '-> 3' nuclease activity of the DNA polymerase. When the donor and acceptor fluorophores separate, the donor fluorescence can be detected at its specific wavelength. In this way, the amplification of the viral RNA and IC can be monitored as the reaction proceeds. CFX96 detects the target sequence during amplification and consists of a fluorometer for the detection of fluorescence during the cycling process. The fluorescent data are displayed on a computer screen, connected to an RT-PCR device, in the form of a graph through specific software. The data were analyzed and reported in the ct-value (threshold cycle), which is the number of PCR cycles taken from a sample to reach the threshold level. A baseline threshold of 25 was defined for FAM fluorescent dye (HCV RNA), and a threshold of 100 was defined for JOE fluorescent dye (IC). Table 1 shows the interpretation of the RT-PCR results.

**Table 1:** Interpretation of the real-time polymerase chain reaction (RT-PCR) results (*) Hypothesis of RT-PCR inhibition or RNA degradation in one of the procedure steps; (**) Possible contamination; (#) In the absence of the IC signal, the sample results cannot be confirmed; therefore, a sample run should be repeated. RT-PCR: Real-time polymerase chain reaction; HCV: Hepatitis C virus; RNA: Ribonucleic acid

Sample	FAM Ct	JOE Ct	Interpretation
Negative Control	Absent	Present	Valid
	Absent	Absent	Not Valid*
	Present	Present	Not Valid**
Positive Control	Present	Absent	Valid
	Absent	Absent	Not Valid*
If the test is valid as per the control			
Samples	Present	Present	HCV RNA Detected
	Absent	Present	HCV RNA not Detected
	Present	Absent	Not Valid^#^
	Absent	Absent	Not Valid^#^

LiPA

The samples, which showed amplification in RT-PCR, were further processed for genotyping of HCV by reverse hybridization strip assay (i.e., LiPA). The GEN-C 2.0 reverse hybridization strip assay from NLM Diagnostics was used for HCV genotyping. The IC was endogenous (mRNA of the housekeeping gene glyceraldehyde 3-phosphate dehydrogenase (GAPDH) and was co-extracted and co-amplified in order to perform the complete procedure. Positive and negative controls were also added with each run. The assay distinguishes between HCV genotypes based on differences in the 5'-UTR and core regions. The test used amplicons with NLM codes AA910/48 or AA896/48. For the most effective outcome, we utilized samples with a viral load of 5x103 to 8x106. Based on the in silico sequence analysis, the device can differentiate between genotypes 1, 2, 3, 4, and 6, as well as subtypes 1a, 1b, 2a/2c, 2b, 3a, 3b, 3c, 3k, 4a, 4b, 4c/4d, 4e, 4f, 4h, 5a, 6a/6b, 6g,f,q, 6m, and 7a. The findings of this assay should be regarded as an aid in deciding the type and duration of antiviral medication for patients with chronic HCV. For result interpretation, an interpretation chart for the reverse hybridization strip assay provided with the kit was used.

Statistical analysis

The data were analyzed using Statistical Product and Service Solutions (SPSS, version 27.0; IBM SPSS Statistics for Windows, Armonk, NY). A chi-square test was performed for the analysis of variables. A P-value of 0.05 or less was considered statistically significant.

## Results

In this study, 109 patients were enrolled who were undergoing maintenance hemodialysis. Out of 109 patients, 64 (58.7%) were male, and 45 (41.3%) were female. The age range of participants was 11-88 years, with mean and standard deviation (SD) of 46.75 and 16.35 years, respectively. The majority of the patients were between 21 and 60 years of age. Of the total patients enrolled, 60 (55.05%) patients belonged to rural backgrounds. Regarding the major risk groups, occupational exposure was among 20 (18.34%), intravenous drug (IVD) use in 14 (12.84%), tattooing in 11 (10.09%), blood transfusion in 85 (77.98%), surgery in 41 (37.61%), and family history for hepatitis infection in immediate family member in 22 (20.18%) patients, as per Table 2.

**Table 2:** Patient characteristics and distribution of anti-HCV antibodies, HCV RNA, and HCV genotypes *p-value considered statistically significant at ≤0.05 The data have been represented as N (i.e., number of patients) and % (i.e., percentage). IVD: Intravenous drug; HCV: Hepatitis C virus; RNA: Ribonucleic acid; Anti-HCV Ab: Anti-hepatitis C virus antibody

Patient Particulars	Anti-HCV Ab-Positive (N=39, 35.78%)	Anti-HCV Ab-Negative (N=70, 64.22%)	P value	HCV RNA Positive (N=24, 22.01%)	HCV RNA Negative (N=85, 77.98%)	P value	HCV Genotype (N=24, 22.01%)		
							1a (N=21, 87.5%)	3b (N=1, 4.2%)	Inconclusive (N=2, 8.3%)
Gender									
Male (N=64, 58.7%)	23	41	0.967	13	51	0.608	10	0	1
Female (N=45, 41.3%)	16	29		11	34		11	1	1
Age group (years)									
<20 (N=3, 2.75%)	1	2	0.077	0	3	0.060	1	0	0
21-40 (N=43, 39.45%)	13	30		8	35		7	0	1
41-60 (N=42, 38.53%)	21	21		14	28		11	1	1
>60 (N=21, 19.27)	4	17		2	19		2	0	0
Residence									
Rural (N=60, 55.05%)	17	43	0.072	7	53	0.003*	6	0	1
Urban (N=49, 44.95%)	22	27		17	32		15	1	1
Duration of Dialysis (months)									
≤12 (N=46, 42.20%)	8	38	<0.0001*	4	42	<0.0001*	4	0	0
>12-36 (N=51, 46.80%)	19	32		11	40		9	0	2
>36 (N=12, 11%)	12	0		9	3		8	1	0
Occupational Exposure									
Yes (N=20, 18.3%)	8	12	0.663	7	13	0.121	6	0	1
No (N=89, 81.7%)	31	58		17	72		15	1	1
IVD Use									
Yes (N=14, 12.84%)	5	9	0.995	1	13	0.150	1	0	0
No (N=95, 87.16%)	34	61		23	72		20	1	2
Surgery									
Yes (N=41, 37.61%)	12	29	0.270	10	31	0.642	9	1	0
No (N=68, 62.84%)	27	41		14	54		12	0	2
Tattooing									
Yes (N=11, 10.09%)	1	10	0.051	1	10	0.275	1	0	0
No (N=98, 89.91%)	38	60		23	75		20	1	2
Family History									
Yes (N=22, 20.18%)	11	11	0.119	8	14	0.069	8	0	0
No (N=87, 79.82%)	28	59		16	71		13	1	2
Number of Blood Units Transfused									
0 (N=24, 22.02%)	11	13	0.576	8	16	0.435	8	0	0
1-5 (N=59, 54.13%)	21	38		11	48		9	1	1
6-10 (N=14, 12.84)	4	10		2	12		1	0	1
>10 (N=12, 11.01%)	3	9		3	9		3	0	0

In our study, the total number of patients who accounted to be seropositive for anti-HCV antibodies was 39 (35.8%) at the end of the study, among which 20 (18.3%) were previously declared positive at the time of screening and 19 (17.4%) were revealed to be positive during the follow-up period. The results of RT-PCR of 5’UTR and core region of HCV demonstrated 24 (22.0%) cases positive for HCV RNA on completion of the study, out of which 10 (9.2%) cases were previously detected seropositive during the screening phase and 14 (12.8%) cases were those which tested positive during the follow-up period.

Out of 109 participants, 70 (64.2%) patients remained uninfected by HCV during the course of the present study where no antibodies were detected by ELISA or no viral RNA detected by RT-PCR. There were 15 patients (10 at screening and five later during the course of the study, where HCV RNA remained undetected by RT-PCR, but anti-HCV antibodies were tested positive by ELISA. The prevalence rate of HCV infection at the beginning of the hemodialysis (screening phase) was 18.3% (20/109 patients), and during the follow up was 17.4% (19/109).

The results after performing LiPA, demonstrated the predominance of HCV genotype 1a noted in 21 (87.5%) patients, followed by genotype 3b detected in one (4.2%) patient only. The result of the HCV genotype was observed to be inconclusive for two (8.3%) patients. The seroprevalence of anti-HCV antibodies was noted to be 35.78%, and the prevalence of HCV RNA was noted to be 22.01%. The incidence of anti-HCV antibodies was 21.34% (19/89), and for HCV RNA, it was 14.14% (14/99) in this study. On observation of the results of this study, the existence of a significant correlation was witnessed between HCV RNA positivity and area of residence (p=0.003). For the following two factors (i.e., anti-HCV antibody and HCV RNA positivity), a statistically significant correlation was established with increasing duration of dialysis in our study. The associated major risk factors, viz. occupational exposure, intravenous drug (IVD) use, surgery, tattooing, family history, or blood unit transfusion, were not seen to be significantly associated with acquiring HCV infection, as shown in Table 2.

Among hemodialysis patients in our study, the most recurring etiological cause for the progression of CKD was noted to be hypertensive nephropathy (69, 63.3%), followed by diabetes mellitus (23, 21.1%), nephrolithiasis (17, 15.6%), autosomal dominant polycystic kidney disease (AD PCKD; 6, 5.5%), and cortical cyst (5, 4.6%). A significant correlation was displayed among hypertensive nephropathy patients and anti-HCV antibody (p<0.0001) and HCV RNA positivity (p<0.0001). The HCV RNA positivity also manifested a significant link in patients with cortical cysts, as evident from Table 3.

**Table 3:** Correlation of patients’ clinical and laboratory parameters with HCV positivity *p-value considered statistically significant at ≤0.05. The data have been represented as N (i.e., number of patients) and % (i.e., percentage). HCV: Hepatitis C virus; RNA: Ribonucleic acid; Anti-HCV Ab: Anti-hepatitis C virus antibody; AD PCKD: Autosomal dominant polycystic kidney disease; CAD-TVD: Coronary artery disease - triple vessel disease; COPD: Chronic obstructive pulmonary disease; AR: Aortic regurgitation; ARDS: Acute respiratory distress syndrome; MODS: Multiple organ dysfunction syndrome; CKD: Chronic kidney disease; ARF: Acute renal failure; B/L: Bilateral

Patients’ Clinical and Laboratory Parameters	Anti-HCV Ab-Positive (N=39, 35.78%)	Anti-HCV Ab-Negative (N=70, 64.22%)	P value	HCV RNA Positive (N=24, 22.01%)	HCV RNA Negative (N=85, 77.98%)	P value	HCV Genotype (N=24, 22.01%)		
							1a (N=21, 87.5%)	3b (N=1, 4.2%)	Inconclusive (N=2, 8.3%)
Etiology of CKD									
Hypertensive nephropathy (N=69, 63.3%)	36	33	<0.0001*	23	46	0.0001*	20	1	2
Diabetes mellitus (N=23, 21.1%)	10	13	0.384	8	15	0.096	7	0	1
Nephrolithiasis (N=17, 15.6%)	3	14	0.089	2	15	0.267	2	0	0
AD PCKD (N=6, 5.5%)	4	2	0.105	3	3	0.089	3	0	0
Cortical cyst (N=5, 4.6%)	3	2	0.246	3	2	0.035*	3	0	0
Obstructive uropathy (N=3, 2.8%)	0	3	-	0	0	-	0	0	0
Acute pyelonephritis (N=3, 2.8%)	0	3	-	0	0	-	0	0	0
MODS (N=2, 1.8%)	1	1	0.674	1	1	0.337	1	0	0
Urinary reflux syndrome (N=2, 1.8%)	1	1	0.674	1	1	0.337	1	0	0
B/L small kidney (N=1, 0.92%)	0	1	-	0	1	-	0	0	0
Tubular necrosis (N=1, 0.92%)	0	1	-	0	1	-	0	0	0
Nephrosclerosis (N=1, 0.92%)	0	1	-	0	1	-	0	0	0
ARF (N=1, 0.92%)	0	1	-	0	1	-	0	0	0
B/L hydronephrosis (N=1, 0.92%)	0	1	-	0	1	-	0	0	0
B/L nephrocalcinosis (N=1, 0.92%)	0	1	-	0	1	-	0	0	0
Unknown etiology (N=14, 12.8%)	3	11	0.23	1	13	0.149	1	0	0
Underlying Co-morbidities									
Pleural effusion (N=9, 8.3%)	2	7	0.373	2	7	0.984	2	0	0
Atrio-ventricular sclerosis (N=6, 5.5%)	1	5	0.317	0	6	-	0	0	0
Trivial or mild AR (N=4, 3.7%)	1	3	0.645	0	4	-	0	0	0
Hepatomegaly (N=4, 3.7%)	2	2	0.548	2	2	0.167	2	0	0
Ischemic heart disease (N=3, 2.8%)	1	2	0.928	1	2	0.631	1	0	0
Tuberculosis (N=3, 2.8%)	3	0	-	1	2	-	1	0	0
Ascites (N=3, 2.8%)	2	1	0.258	2	1	0.058	2	0	0
CAD-TVD (N=2, 1.8%)	0	2	-	0	2	-	0	0	0
Psychiatric patient (N=2, 1.8%)	0	2	-	0	2	-	0	0	0
ARDS (N=1, 0.92%)	0	1	-	0	1	-	0	0	0
Cholelithiasis (N=1, 0.92%)	0	1	-	0	1	-	0	0	0
COPD (N=1, 0.92%)	0	1	-	0	1	-	0	0	0
Duodeno-jejunal stent (N=1, 0.92%)	0	1	-	0	1	-	0	0	0
Gall bladder sludge (N=1, 0.92%)	0	1	-	0	1	-	0	0	0
Hyperthyroidism (N=1, 0.92%)	0	1	-	0	1	-	0	0	0
Hypothyroidism (N=1, 0.92%)	0	1	-	0	1		0	0	0
Postpartum sepsis (N=1, 0.92%)	0	1		0	1		0	0	0
Prostate carcinoma (N=1, 0.92%)	0	1	-	0	1	-	0	0	0
Prostatomegaly (N=1, 0.92%)	0	1	-	0	1	-	0	0	0
Thimble bladder (N=1, 0.92%)	0	1	-	0	1	-	0	0	0
Typhoid (N=1, 0.92%)	0	1	-	0	1	-	0	0	0
Other Complications									
Renal amylodosis (N=1, 0.92%)	0	1	-	0	1	-	0	0	0
Uremic encephalopathy (N=1, 0.92%)	0	1	-	0	1	-	0	0	0
Blood Picture									
Anemia (N=43, 39.5%)	7	26	0.036*	4	29	0.101	3	0	0
Neutrophilia (N=9, 8.3%)	2	7	0.373	1	8	0.412	1	0	0
Lymphopenia (N=5, 4.6%)	0	5	-	0	5	-	0	0	0
Anisocytosis (N=5, 4.6%)	2	3	0.841	1	4	-0.912	1	0	0
Leukocytosis (N=4, 3.7%)	0	4	-	0	4	-	0	0	0
Microcytosis (N=2, 1.8%)	1	1	0.674	1	1	-0.337	1	0	0
Thrombocytopenia (N=1, 0.92%)	0	1	-	0	1	-	0	0	0

## Discussion

HCV infection is a significant public health concern due to the lack of an effective vaccine and the increased risk of serious complications [1]. Consequently, prevention and early detection of HCV are imperative. One of the primary concerns regarding HCV transmission is its potential for nosocomial spread. In particular, dialysis units are critical environments where stringent universal precautions are necessary. In this study, 58.7% of patients were males, and 41.3% were females, demonstrating a male preponderance. A similar pattern of admission in the HD ward was noted in studies conducted by Roy et al. [14], but male predominance in the dialysis ward has been shown by Madhavan et al. [4]. The age of study participants ranged from 11 to 88 years in our study. The mean age was calculated to be 46.75 years with an SD of 16.35 years. A similar age range was noted in a study by Halle et al. [15].

In the majority of studies, data on rural and urban distribution of patient populations are either unavailable or found to be statistically insignificant in relation to HCV seropositivity. Our study shows a higher rural population (55.04%) in contrast to the urban population. The above finding in our study is in disagreement with a study conducted in Egypt by Kerollos et al., which shows a higher urban population (i.e., 54%) [16]. The anti-HCV antibodies, as well as HCV RNA, were detected more in urban study participants compared to rural patients. The HCV RNA results in both populations were compared and found statistically significant. The reasons for the higher prevalence of HCV infection in urban populations could be the higher rate of HCV distribution among the general urban population or sexual activities, tattooing, IV drug users, etc. Urban areas also have higher population densities and more concentrated healthcare facilities, so such populations always have a greater chance of encounters between infected and susceptible individuals. The etiology of end-stage renal disease (ESRD) in our study was hypertensive nephropathy among 69 (63.3%), followed by diabetes mellitus in 23 (21.1%) and nephrolithiasis in 17 (16.5%). Our findings correlate well with the study conducted by Kataruka et al., which also displays hypertensive nephropathy as the most recurring etiological cause [17].

In contrast to the above observations in our study, various other studies depict diabetic nephropathy to be the most frequent etiological cause of ESRD [18]. Both diabetes and hypertension are related to CKD. The inadequate blood flow in hypertension leads to tubular atrophy, interstitial fibrosis, and glomerular alterations, which in turn lead to kidney failure at later stages. The prevalence of hypertension in India is increasing steadily and recorded at 30.7% by the Cardiological Society of India during the pan India blood pressure camp [19]. HCV and CKD are interrelated as patients with CKD are at risk of acquiring HCV infection during hemodialysis, while HCV infection causes excess cryoglobulin production, which may initiate immune complex-mediated vasculitis, inducing vascular thrombosis and inflammation. HCV infection increases further CKD risk and progression of ESRD due to unknown mechanisms [20]. In the present study, the mean and SD of the duration of dialysis were observed to be 22.86±18.69 (months), but a high variation was observed in other studies, which ranged from 6.03 to 79.78 months [4,14]. The HD vintage in the majority of the patients (46.79%) was noted to be ≥ 12-36 months. Similar results, which agreed with our results, have been reported in Indian studies conducted by Madhavan et al. [4] and Kalita et al. [21] and in international studies performed by Halle et al. [16].

Meanwhile, studies conducted in the Middle East region reveal that the majority of the patients had longer HD vintage of >36 months [22,23]. The maximal number of cases (54.1%) in the present study had one to five units of blood transfusion. This finding was in concordance with previous studies conducted worldwide by Zamani et al. and Khan et al. [24,25], but a study conducted in Spain by Forns et al. shows a comparatively higher number of blood units transfused in the HD population [26]. Family history of HCV infection in family members was found to be 22/109. Out of these 22 cases, 11 patients (50%) were recorded seropositive for anti-HCV antibodies in the current study. The transmission of HCV by unsafe sexual activity, as well as vertically from mother to baby, is well known. Since sexual transmission accounts for 15%, the sharing of shaving razors or blades as a route of transmission is around 14.5%, and body piercing around 30.1%; hence, it is well correlated with HCV seroprevalence and infection [27]. The rate of incidence and prevalence of HCV infection among HD patients differs markedly 254 from country to country and also among various dialysis units within a single country [28,29]. Among the total 19 novel seroconverted cases detected by ELISA, 14 cases were found to be HCV RNA positive, leading to discordance between anti-HCV Ab (antibody) and HCV RNA positivity in our study, indicating current viral infection in these 14 patients and depicting the persistence of antibody even after diminished viral load. This discordant correlation is supported by international studies performed by Silva et al. who showed 92 (73.6%) of 125 anti-HCV-positive patients to be HCV RNA positive [30]. The incidence of HCV RNA in our study was found to be 14.14% (14/99). This finding was in concordance with previous other studies by Kerollos et al. (13.2%) [16].

However, few works conducted in France by Somi et al. [31] show a very low HCV RNA incidence rate (i.e., 3.95%), whereas a study conducted in Iran by Al-Rubaie et al. depicts a very high incidence rate (i.e., 40.3%) [32]. The cases of anti-HCV Ab-positive, but RNA-negative, individuals had either attained sustained viral response (SVR) and viral clearance following treatment or undetectable HCV replication [33]. The false-positive antibody detection has also been reported to be associated with ethnicity, age, raised erythrocyte sedimentation rate, autoantibodies, and patients with prosthetic devices [34]. The 14 HCV RNA-positive patients have acquired HCV infection during the course of study or hemodialysis. The exact reason is not known as there are multiple risk factors associated such as the use of unsterilized injection needles, total transfused blood volume, multiple blood transfusions, lack of proper enforcement of universal precautions, prolonged vascular access, exposure to infected patients, contaminated equipment, and breach in infection control practices such as proper cleaning and disinfection of equipment and environmental surfaces, hand hygiene and PPE, high co-morbid illness burden, and greater frequency of interventions [2]. Blood transfusion, however, continues to be a probable risk factor for HCV infection acquired via HD due to low levels of seroprevalence detected in blood donors ranging from 0.39% to 5.1% in India [35,36].

Although no statistically significant association was witnessed, HCV may have been acquired through blood transfusion. It is also noteworthy that, even though at the majority of centers including ours, blood and blood products required for transfusion are diligently screened for anti-HCV antibody testing by ELISA. The absence of antibodies cannot be completely ruled out in case of HCV infection [15,37]. Multiple studies conducted in the northern and eastern regions of India found a predominance of HCV genotype 3, followed by HCV genotype 1 infection [38], as compared to equal distribution of HCV genotypes 1 and 3 in the southern and western regions of India [38,39]. In our study, the HCV genotype for all HCV RNA-positive cases was detected by performing LiPA. Surprisingly, in our study, we noted that, of the total 24 HCV RNA-positive HD patients, 21 (87.5%) cases were detected to carry HCV genotype 1a. One (4.2%) case was observed to be HCV genotype 3b. The HCV genotype of the remaining two (8.3%) cases was noted to be inconclusive. The genotyping test may remain inconclusive due to multiple factors, such as the presence of potential cross-reactivity markers (e.g., HBV, HIV, HPV, etc.) or the presence of any potentially interfering substance or even due to sample handling and processing errors (e.g., cross-contamination of PCR products) or whole system PCR failure. The selective preponderance of HCV genotype 1a in HD patients mirrored a similar pattern to the work conducted by Roy et al. [14] who demonstrated HCV genotype 1a to be 54.1%. The predominance of HCV genotype 1a in the current study indicates the presence of this genotype in populations of Rajasthan, especially in and around the study center as there is no published data on the same from this part of the country. Another reason for the predominance of HCV genotype 1a could be person-to-person transmission in the hemodialysis unit, which was also suggested by Roy et al. [14]. Although with the advent of pan-genotypic HCV treatment regimens, HCV genotyping is no longer required when the patient is suitable for simplified treatment regimens; however, pre-treatment genotyping is recommended in patients who had evidence of cirrhosis and or past unsuccessful HCV treatment [40].

Limitations

It is crucial to acknowledge a few limitations of our study. In the study, all the samples that were ELISA seronegative for anti-HCV antibodies were pooled (five samples each) and then processed via rRT-PCR for RNA detection if the availability of serotyping kits is limited. Additionally, the study had a small sample size and was conducted on a small scale, so the prevalence of HCV genotype and sub-types detected in patients on hemodialysis cannot be specified for the general population as it included patients attending tertiary care hospitals associated with Dr. Sampurnanand Medical College, Jodhpur, only.

## Conclusions

Identification of HCV infection via a nucleic acid-based molecular assay such as RT-PCR has been proven to be of significant clinical benefit. It is concluded from the study that screening via enzyme immunoassays (EIA) such as ELISA might not prove to be suitable in HD units, among which HCV infection is prevalent as a consequence of false-negative results. It was also established as a conclusion of our study that knowledge of the geographical distribution of circulating HCV genotypes and sub-types is imperative in the improvisation of clinical management of dialysis patients as the effectiveness of treatment is also found to be genotype-dependent, and it will also provide an aid in future vaccine formulations. In line with the recommendations by the Kidney Disease Outcome Quality Initiative (KDOQI) guidelines, emphasis should be laid on enforcement of the quality control and quality assurance measures covering entire dialysis units by healthcare professionals across the state, including intermittent serological testing for the prevalence of anti-HCV antibodies among complete dialysis staff.
